# Combined Bacteriophage and Antibiotic Treatment Prevents *Pseudomonas aeruginosa* Infection of Wild Type and *cftr*- Epithelial Cells

**DOI:** 10.3389/fmicb.2020.01947

**Published:** 2020-08-26

**Authors:** Alexandre Luscher, Juliette Simonin, Léna Falconnet, Benoît Valot, Didier Hocquet, Marc Chanson, Grégory Resch, Thilo Köhler, Christian van Delden

**Affiliations:** ^1^Transplant Infectious Diseases Unit, Geneva University Hospitals, Geneva, Switzerland; ^2^Department of Microbiology and Molecular Medicine, University of Geneva, Geneva, Switzerland; ^3^Department of Pediatrics, Gynecology and Obstetrics, Geneva University Hospitals and University of Geneva, Geneva, Switzerland; ^4^Department of Cell Physiology and Metabolism, University of Geneva, Geneva, Switzerland; ^5^UMR CNRS 6249 Chrono-Environnement, University of Franche-Comté-Bourgogne, Besançon, France; ^6^Bioinformatique et Big Data au Service de la Santé, UFR Santé, Université de Bourgogne Franche-Comté, Besançon, France; ^7^Department of Infection Control, University Hospital of Besançon, Besançon, France; ^8^Department of Fundamental Microbiology, University of Lausanne, Lausanne, Switzerland

**Keywords:** bacteriophage, *Pseudomonas aeruginosa*, epithelial cell infection, cystic fibrosis, antibiotic resistance

## Abstract

With the increase of infections due to multidrug resistant bacterial pathogens and the shortage of antimicrobial molecules with novel targets, interest in bacteriophages as a therapeutic option has regained much attraction. Before the launch of future clinical trials, *in vitro* studies are required to better evaluate the efficacies and potential pitfalls of such therapies. Here we studied in an *ex vivo* human airway epithelial cell line model the efficacy of phage and ciprofloxacin alone and in combination to treat infection by *Pseudomonas aeruginosa*. The Calu-3 cell line and the isogenic CFTR knock down cell line (*cftr*-) infected apically with *P. aeruginosa* strain PAO1 showed a progressive reduction in transepithelial resistance during 24 h. Administration at 6 h p.i. of single phage, phage cocktails or ciprofloxacin alone prevented epithelial layer destruction at 24 h p.i. Bacterial regrowth, due to phage resistant mutants harboring mutations in LPS synthesis genes, occurred thereafter both *in vitro* and *ex vivo*. However, co-administration of two phages combined with ciprofloxacin efficiently prevented PAO1 regrowth and maintained epithelial cell integrity at 72 p.i. The phage/ciprofloxacin treatment did not induce an inflammatory response in the tested cell lines as determined by nanoString^®^ gene expression analysis. We conclude that combination of phage and ciprofloxacin efficiently protects wild type and *cftr*- epithelial cells from infection by *P. aeruginosa* and emergence of phage resistant mutants without inducing an inflammatory response. Hence, phage-antibiotic combination should be a safe and promising anti-Pseudomonas therapy for future clinical trials potentially including cystic fibrosis patients.

## Introduction

*Pseudomonas aeruginosa* is an opportunistic pathogen, able to cause acute infections as well as chronic infections in cystic fibrosis and immuno-compromised patients (Cohen and Prince, 2012). Designing optimal treatment regimens against this organism remains a challenge, due to its intrinsic antibiotic resistance and its ability to develop and acquire additional antibiotic resistance determinants (Li et al., 2015; Botelho et al., 2019). Due to the lack of novel antibiotics, alternative treatment strategies are urgently needed. One option is bacteriophages (Twort, 1915; d’Hérelle, 1917), which are natural predators of bacteria. Their use as antimicrobials predates the antibiotic era. Among them, lytic phages have been used for many years in the Eastern European countries, mainly as a topical treatment against bacterial infections (Kutateladze and Adamia, 2010; Myelnikov, 2018). The possibility to treat efficiently multidrug resistant (MDR) bacteria with phages has recently spurred renewed interest in this field (Gordillo Altamirano and Barr, 2019). Phages present several advantages over conventional antibiotics, the major one being their specificity, since they usually target a single bacterial species, while leaving the host microbiota unaffected. Secondly, phage replication is dependent on the presence of the host-bacterium and is therefore self-limiting. The main concerns for therapeutic use of phages are the possibility to transfer virulence or antibiotic resistance genes, requiring in depth analysis of the phages’ genomes (Torres-Barcelo, 2018). A drawback that phages share with antibiotics is emergence of phage-resistant mutants, resulting mainly from the loss of phage receptor structures located on the bacterial surface (lipopolysaccharide, pili, outer membrane proteins), which are not always essential for bacterial survival. However, administration of phage cocktails or their combination with antibiotics could potentially circumvent this problem (Vandenheuvel et al., 2015; Tagliaferri et al., 2019).

So far, phage treatments have been tested in a short time scale (<24 h) on cell cultures and in various invertebrate and mammalian pre-clinical models (Heo et al., 2009; Alemayehu et al., 2012; Saussereau et al., 2014; Waters et al., 2017; Trend et al., 2018). However, the emergence of resistance upon longer incubation times (>24 h) and the effect on host inflammatory pathways has been studied only for *S. aureus* and *E. coli* phages (Freyberger et al., 2018; Dufour et al., 2019). Here, we investigated the effect of phage/antibiotic treatments on polarized human airway epithelial cells infected with *P. aeruginosa*. We found that wild type and *cftr*- mutant cell lines infected with *P. aeruginosa* can be efficiently treated with phages. However, prevention of emergence of phage resistant mutants beyond 24 h required simultaneous administration of phages and antibiotic. Using RNA expression analyses, we further showed that phages and antibiotics *per se* did not induce a pro-inflammatory response in epithelial cells.

## Materials and Methods

### Bacterial Strains, Phages and Growth Conditions

Strains used and generated in this study are listed in Table 1. *P. aeruginosa* was grown in lysogeny broth (LB) at 37°C with agitation (240 rpm). Phage vB_PaeP_4024 (ϕ24) is a N4-like phage, closely related to the lytic phage PEV2 (KU948710.1). Phage vB_PaeP_4054 (ϕ54) is a LUZ24-like phage, closely related to the lytic phage TL (HG518155.1). These two phages were isolated from the commercially available PYO BACTERIOPHAGE preparation developed by the Eliava Institute (Tbilisi, Georgia) and belong to the podoviridae family. The phage vB_PaeS_4069 (ϕ69) belongs to the siphoviridae family and was kindly provided by Christine Pourcel (University of Paris-Sud, France). All phages yielded clear plaques on *P. aeruginosa* strain PAO1 and showed different host ranges when tested on a collection of more than 100 *P. aeruginosa* clinical isolates mostly collected at the University Hospital of Lausanne (CHUV, Supplementary Table S3).

**TABLE 1:** Antibiotic and phage susceptibility of phage-selected PAO1 mutants.

		MICs (mg/L)			Susceptibility to phage						
Strain	Selection	CAZ	CIP	GEN	COL	AZT	ϕ24	ϕ54	ϕ69	Genotype	Phenotype
PAO1	NA	1	0.25	0.5	1	128	S	S	S	wild type	
PAO1-R1.1*	ϕ54 (*in vitro*)	2	0.06	0.25	0.25	8	R	R	R	*galU,hisF2* (T20I)	pyomelanin
PAO1-R1.4	ϕ54 (*in vitro*)	0.5	0.03	0.5	0.25	4	R	R	R	*algC*241Δ2bp	
PT2426	ϕ24/69/CIP	2	0.25	0.5	1	256	R	S	S	ND	mucoid
PT2427	ϕ24/69/CIP	1	0.25	0.5	0.5	16	R	R	R	*galU*	pyomelanin

NA, not applicable; S, susceptible to phage; R, resistant to phage; CAZ, ceftazidime; CIP, ciprofloxacin; GEN, gentamicin; COL, colistin; AZT, azithromycin. *PAO1-R1.1 carries a 40 kb chromosomal deletion, including *galU* and the *mexXY* efflux pump operon.

### Cell Culture

Human airway epithelial Calu-3 WILD TYPE cells (HTB-55TM) were purchased from the American Type Culture Collection (ATCC, Manassas, VA, United States). Calu-3 *cftr*- and Calu-3 CTL, are isogenic cell lines generated by CRISPR-Cas9, carrying respectively a CFTR knock down and a scramble RNA construct (Bellec et al., 2015). Cells were cultivated at 37°C in 5% CO_2_ in Minimum Essential Medium (MEM) supplemented with 10% decomplemented Fetal Bovine Serum (FBS), 1% non-essential amino acids, 1% sodium pyruvate and 1% penicillin/streptomycin and 0.25 μg/mL amphotericin B (Fungizone). All reagents, except FBS, were purchased from Life Technologies (Saint-Aubin, France). Well-polarized monolayers of Calu-3 cells were obtained by seeding 175,000 cells onto 0.33-cm^2^ porous (0.4 μm) Transwell polyester inserts (Transwell 3470, Corning Life Sciences, Hazebrouck, France). The inserts were submerged and cultured for 5 days until cells reached confluence. Cell differentiation was induced by culturing cells at the air-liquid interface for at least 15 days for the Calu-3 CTL and Calu-3 *cftr*- epithelial cells and for at least 10 days for the Calu-3 WILD TYPE cells. Penicillin, streptomycin and amphotericin B (Fungizone) were removed from the cells 24 h before bacterial infection. Secreted air-surface liquid was removed from the cells prior to infection to allow the release of residual antibiotics from the cells.

### Cell Infection and Antibacterial Treatments

5 × 10^5^ Calu-3 cells were polarized on Transwell filters for more than 10 days and subsequently infected as described before (Losa et al., 2015). Briefly, an overnight culture of *P. aeruginosa* PAO1 was washed and resuspended in saline buffer (NaCl 0.9%, HEPES 10 mM, CaCl_2_ 1.2 mM) to a density of 10^5^ CFU/ml, and 10 μl of this suspension was added apically to the Transwell filter (i.e., final inoculum of 10^3^ ± 10^1^ CFUs, MOI of 0.002). Ten μl of saline buffer was added on uninfected controls. Cells were incubated for 6 h at 37°C in 5% CO_2_ atmosphere, before treatment was applied: 10 μl of saline buffer containing 10^3^ ± 10^1^ PFUs of each phage and/or ciprofloxacin at 4 μg/ml final concentration in the apical fluid. Ten μl of saline buffer was added on untreated controls, and cells were incubated as above for further until 24 or 72 h post-infection (p.i.). At each time point, *trans-*epithelial resistance was measured and CFU and PFU counts were performed.

### Determination of Viable Bacteria and Infective Phage Particles

To determine the CFU and PFU counts in the wells, 200 μl of saline buffer was added apically to the Transwell filter and 100 μl of this wash were recovered. Ten-fold serial dilutions were performed in saline buffer and 5 μl of each dilution were spotted on LB-agar plates (for CFU count) and on a soft-agar PAO1 lawn (for PFU count). To quantify the individual phages in experiments performed with cocktails, dilutions of the apical wash were also spotted on a PAO1 mutant resistant to ϕ24 but susceptible to ϕ69.

### Whole Genome Sequencing of Bacterial Isolates

Two isolates surviving single phage treatment were submitted to whole genome sequencing. Bacterial DNA was extracted using the Tissue DNA extraction kit (Qiagen) and sequenced on an Illumina platform (single 100-bp reads with a minimum of 400× genome coverage). After subsampling at 80× coverage, reads were aligned against the PAO1 reference genome (AE004091.2) using bwa mem (v0.7.15, arXiv:1303.3997v1). Variant calling was performed using freebayes (v1.1, arXiv:1207.3907) with haploid mode, a minimum of 10 reads coverage, 20 quality for base call and map and 0.8 alternated fraction. Deletion of gene blocks was searched using featureCounts (v1.6.3) with a minimum overlap of 20 (Liao et al., 2014).

### *Trans-*Epithelial Resistance (TER) Measurements

*Trans*-epithelial resistance was measured using chopstick electrodes as described previously (Srinivasan et al., 2015). Briefly, at the indicated post-infection time points the Transwell filters were transferred into a new 24-well plate containing 600 μl of saline buffer/well. 200 μl of saline buffer were added apically and the electrical resistance was measured in duplicate with a volto-meter (EVOM, World Precision Instruments, Inc). Final TERs were reported using the formula:

$$T\,E\,R=R_{m\,e\,a\,s\,u\,r\,e\,d}\,\left(\Omega \right)\cdot A\,r\,e\,a_{T\,r\,a\,n\,s\,w\,e\,l\,l}\,\left(c\,m^{2}\right)$$

### NanoString Gene Expression Analysis

Total RNA was extracted from epithelial cells on the Transwell filter with the RNeasy kit according to the manufacturer’s guidelines (Qiagen, Germany). The expression of 249 inflammatory genes was measured in uninfected and phage/antibiotic-treated PAO1-infected epithelial cells using the NanoString RNA detection kit (Geiss et al., 2008). Briefly, 100 ng of RNA from Calu-3 cell lines were hybridized for 20 h at 65°C with inflammation pathway probes (nCounter inflammation panel Human v2, NanoString^®^). Post-hybridization washes and bound-RNA loading on the nCounter Prep station were processed following NanoString^®^ guidelines. Sample normalization was performed on six housekeeping genes: GUSB, HPRT1, TUBB, PGK1, CLTC, and GAPDH. A two-fold induction was considered as significant. Purified flagellin protein from *P. aeruginosa* (FLA-PA Ultrapure) was purchased from InvivoGen (United States).

### Statistical Analyses

All graphs and statistical data analyses were performed with GraphPad Prism (version 8). One-way ANOVAs were used to establish the significance of differences observed in the *trans-*epithelial resistance measurements.

## Results

### *P. aeruginosa* Lowers *Trans-*Epithelial Resistance of Wild Type and *cftr*- Calu-3 Cell Lines

**FIGURE 1:**
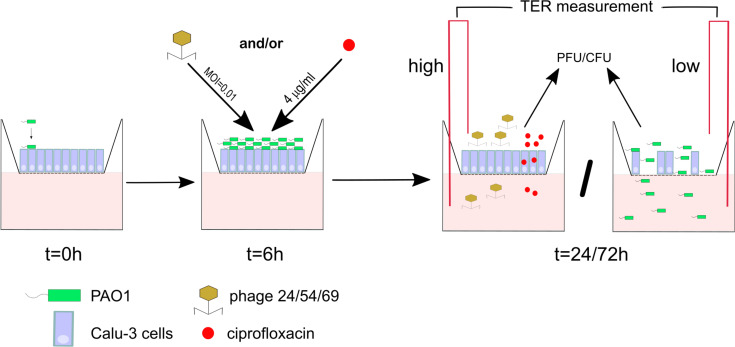
Scheme of the infection and treatment schedule. 1 × 10^3^ bacterial cells are added at *t* = 0 h to a well seeded with 5 × 10^5^ Calu-3 cells. After 6 h incubation PAO1 has reached approximately 10^6^ CFU/well, at which time ciprofloxacin (CIP) or three different phages alone or in combination are added apically. Incubation is continued until 24 or 72 h, at which time point epithelial cell integrity (TER measurement), bacterial load (CFU) and phage titers (PFU) were determined.

**FIGURE 2:**
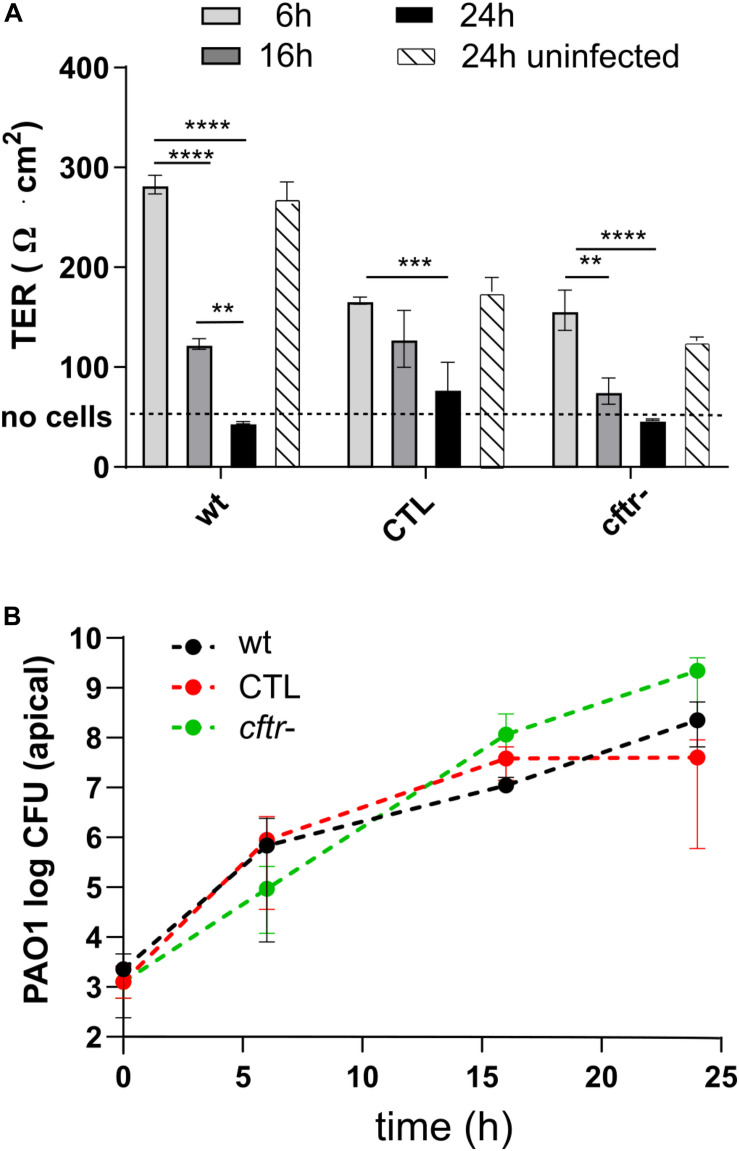
Infection kinetics with *P. aeruginosa* PAO1 on Calu-3 cell lines. **(A)** The wild type, CTL and *cftr-* cell lines were infected at *t* = 0 h with PAO1. The CTL and *cftr-* cell lines showed a lower TER at *t* = 0 h but similar TER values as the wild type Calu-3 cells after 16 and 24 h. **(B)** PAO1 showed similar growth kinetics on the three Calu-3 cell lines. Values represent mean and SEM (panel **A**), and median and range (panel **B**) of three independently performed experiments. Differences in TER between the time points were assessed using a one-way ANOVA; **p < 0.01, ***p < 0.005, ****p < 0.001.

We sought in this study to compare the response of epithelial cell lines to bacteriophages and antibiotics upon infection with *P. aeruginosa* reference strain PAO1. We used three cell lines: (i) the Calu-3 human airway cell HTB-55TM (wild type) (Shen et al., 1994), (ii) an isogenic knock down of the CFTR channel locus (*cftr*-), and (iii) the corresponding control cell line expressing a scramble RNA (CTL) (Bellec et al., 2015). We determined the effect of *P. aeruginosa* infection on cell layer integrity by measuring the *trans-*epithelial resistance (TER), which decreases when breaches in the epithelial layer occur (Figure 1). Apical infection of the three cell lines with 10^3^ PAO1 CFU/well resulted in a time-dependent drop of TER, indicating progressive disruption of the epithelial cell barrier function. At 6 h post-infection (p.i.) the TER values for each cell line were comparable to those of the respective uninfected control values at 24 h, indicative of an intact epithelium. However, at 16 h all cell lines showed significant decreases in TER, while at 24 h, TER values were comparable to those of an empty filter without cells (dotted line in Figure 2A). Visual inspection of the Transwell filters under a light microscope confirmed Disruption of the epithelial layer. To determine if the detached Calu-3 cells 24 h p.i. were still viable, we collected and stained these cells with trypan blue. More than 84% of detached Calu-3 wild type cells were stained by Trypan blue compared to 92% of uninfected Calu-3 cells but treated with lactate dehydrogenase lysis buffer. This indicated that detached cells were mostly dying cells resulting from infection with PAO1 (data not shown).

We next compared the PAO1 apical growth on the Calu-3 wild type, CTL and *cftr*- epithelial layers by CFU counting. PAO1 showed similar growth kinetics on the three cell lines starting from 10^3^ CFU/well and reaching 10^7^ to 10^9^ CFU/well at 24 h p.i. (Figure 2B). The 2-to 3-log increase in bacterial density at 6 h p.i. did not affect TER, while progressive cell layer destruction and cell detachment occurred thereafter for the three cell lines (Figure 2A). We also tested twelve CF-isolates from six patients on the Calu-3 wild type and *cftr*- cells. These isolates caused little or no damage to the epithelial cell layer during 24 h incubation (data not shown). Since this would have limited the evaluation of phages to prevent damage to epithelial cells, we decided to pursue the study with PAO1.

### Treatment of Calu-3 Cells With Mono-Phage or Ciprofloxacin Maintains Epithelial Cell Integrity for 24 h p.i.

**FIGURE 3:**
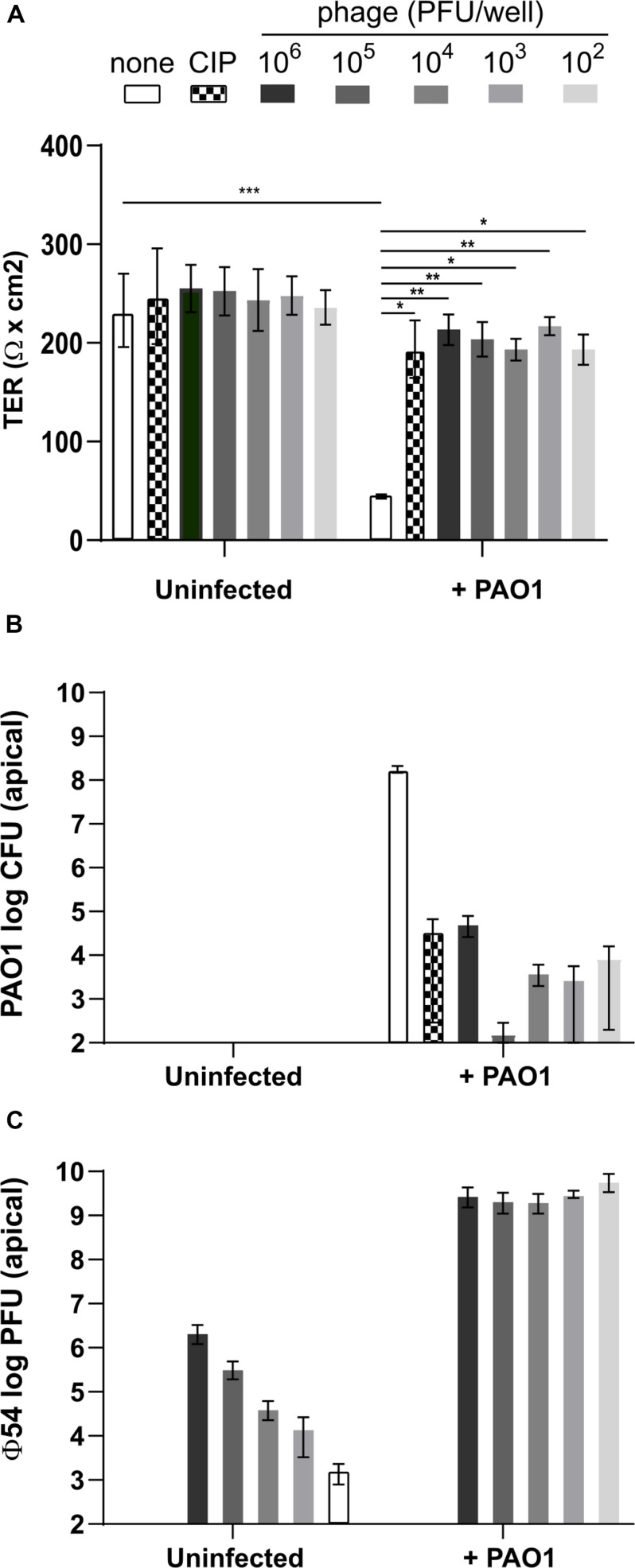
Measurement of *trans-*epithelial resistance (TER), CFU and PFU at 24 h of Calu-3 cells infected with *P. aeruginosa* PAO1 and treated with ϕ54. **(A)** Phages alone do not affect TER and remain infective for 24 h. Cell integrity is maintained by ciprofloxacin (CIP) treatment (4 mg/ml) or addition of phage at an initial inoculum of 10^2^ to 10^6^ PFU/well. **(B)** Treatment with CIP or phage reduces PAO1 counts after 24 h. **(C)** In the absence of PAO1, phage titers remain constant during 24 h. In the presence of PAO1, phage replicate to the same titer independent of the initial inoculum. Values represent median and range of three independently performed experiments. Differences in TER between the conditions were assessed using a one-way ANOVA; *p < 0.05, **p < 0.01, ***p < 0.005.

Since at 6 h p.i. the TER was not affected by *P. aeruginosa* infection, although bacterial cell density had increased to ca. 10^6^ CFU/well, we decided to use this time point to initiate the anti-Pseudomonas treatments. We used three different bacteriophages, vB_Pae_4024 (ϕ24), vB_Pae_4054 (ϕ54) and vB_PaeS_4069 (ϕ69), as well as ciprofloxacin as a control treatment. We added either ϕ54 alone at a multiplicity of infection (MOI) of 10^–4^ to 10^1^ or ciprofloxacin at 4 mg/L (16-fold the MIC in MH broth medium) to the infected cells and determined the TER, CFU and PFU at 24 h p.i. ϕ54 was chosen for the mono-phage treatments since the plaque diameter was larger compared to those of the other phages. Addition of phage or ciprofloxacin, in the absence of PAO1 infection, did not affect TER and the integrity of the cell lines was comparable to the control after visual inspection under the microscope (Figure 3A). Infection with *P. aeruginosa* PAO1 deteriorated cell integrity, as evidenced by the low TER values at 24 h p.i. In contrast, treatment with ciprofloxacin or ϕ54 even at the lowest MOI (10^–4^) preserved Calu-3 cell integrity (Figure 3A). However, ciprofloxacin or phages did not completely eradicate PAO1 cells but led to a drop of CFUs by 4 to 6-logs, compared to the untreated control condition (Figure 3B). Interestingly, in the absence of any bacteria the number of infective phage particles remained stable until the end of the incubation period. In the presence of PAO1, ϕ54 replicated efficiently to reach similar titers at 24 h p.i. (10^9^ PFU/well) independently of the phage inoculum (Figure 3C). No phage particles were detected at the basolateral side of an uninfected Transwell filter (data not shown), suggesting that the epithelium is impermeable to ϕ54.

### PAO1 Escapes Mono-Phage Therapy or Ciprofloxacin Treatment 24 h p.i.

**FIGURE 4:**
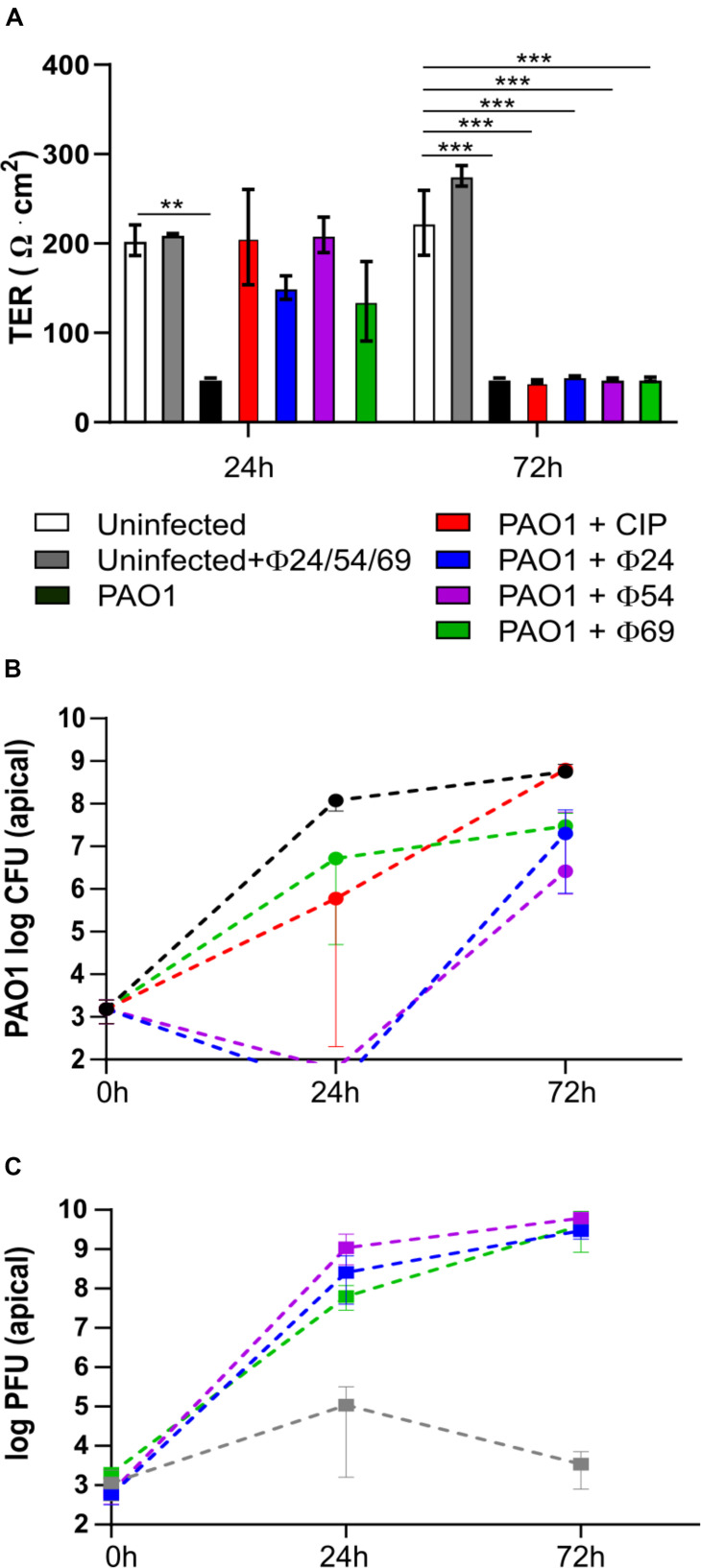
Measurement of *trans-*epithelial resistance (TER), CFU and PFU at 24 and 72 h p.i. of Calu-3 cells infected by *P. aeruginosa* PAO1 and treated by ciprofloxacin (CIP) or phages. **(A)** Single phage or CIP rescued PAO1-infected wild type Calu-3 cells at 24 h but not at 72 h p.i. **(B)** PAO1 viability is reduced at 24 h by phage or CIP but returns to almost untreated control levels after 72 h p.i. **(C)** phage replication increases during 72 h in wells containing PAO1 bacteria. Values represent mean and SEM (panel **A**), and median and range (panels **B,C**) of three independently performed experiments. Differences in TER between the conditions were assessed using a one-way ANOVA; **p < 0.01, ***p < 0.005.

To follow the efficacy of mono-phage therapy beyond 24 h p.i., we further incubated the PAO1-infected Calu-3 cell lines treated with ciprofloxacin or individual phages up to 72 h p.i. At this time point, the TER values had dropped to 50 Ω x cm^2^ for all treatment conditions, indicating disruption of epithelial cell integrity (Figure 4A). Upon treatment with ϕ24 and ϕ54, PAO1 CFUs dropped below detection at 24 h p.i., however regrowth occurred resulting in bacterial densities close to those of the untreated control condition at 72 p.i. (Figure 4B). Phage replication occurred mainly during the initial 24 h, reaching similar titers for the three phages at the end of the experiment (Figure 4C). In the absence of bacteria, phage titers remained stable and phages remained infective after 72 h on the epithelial cells (Figure 4C). We hypothesized that bacterial regrowth occurred due to phage-resistant PAO1 mutants. Two randomly selected colonies surviving ϕ54 treatment were submitted to WGS. One mutant (PAO1-R1.1) produced the brown pigment pyomelanin and carried a 40-kb deletion encompassing the *hmgA* gene (homogentisate-1,2-dioxygenase) and the *galU* gene involved in LPS synthesis (Dean and Goldberg, 2002). An additional mutation occurred in the *hisF2* gene (Thr to Ile substitution at codon 20), embedded in the O5 serotype LPS biosynthesis operon of PAO1. The second mutant (PAO1-R1.4) carried a 2-bp deletion in the *algC* gene, which is required for LPS, alginate and Psl polysaccharide synthesis (Table 1). Both *algC* and *galU* mutants were also resistant to phages ϕ24 and ϕ69, suggesting that the LPS constitutes the receptor for the three phages (Coyne et al., 1994; Dean and Goldberg, 2002). Interestingly, both phage-resistant mutants showed increased susceptibility to ciprofloxacin, azithromycin and colistin (Table 1).

### Phage Cocktail Combined With Antibiotic Prevents PAO1 Regrowth

**FIGURE 5:**
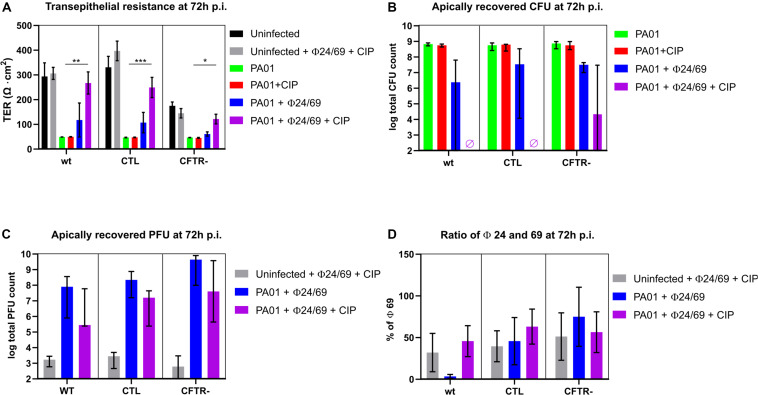
Measurement of *trans-*epithelial resistance (TER), CFU and PFU at 72 h p.i. **(A)** Only combined phage and ciprofloxacin (CIP) treatment protected epithelial cells from PAO1 infection (violet bars). **(B)** No viable bacteria (detection limit of 10^2^ CFU/well) were recovered from the apical side of the wild type and CTL cells, while surviving bacteria occurred in 2/3 experiments on the *cftr*-cells. **(C)** Phages remained at the level of the inoculum in the absence of bacteria (gray bars) but replicated in the presence of PAO1 treated with phages alone (blue bars). CIP reduced phage replication due to killing of PAO1 host cells. **(D)** Phages were added at *t* = 0 h at a 50:50 ratio. With the exception of the wild type cell line (blue bar), this ratio was maintained until 72 h p.i. Values represent mean and SEM (panels **A,D**), and median and range (panels **B,C**) of three independently performed experiments. Differences in TER between the conditions were assessed using a one-way ANOVA; *p < 0.05, **p < 0.01, ***p < 0.005.

We next evaluated whether phage cocktails could prevent the PAO1 regrowth observed at 72 h. We therefore tested a two-phage cocktail ϕ24/ϕ69 prepared at a 1:1 ratio, either alone or in combination with ciprofloxacin. While ciprofloxacin alone did not prevent the drop in TER at 72 h, the phage cocktail alone protected partially all three cell lines. Markedly, when both treatments were combined, TER values were comparable to those of the uninfected control, suggesting complete protection of the epithelial cell layer (Figure 5A). *P. aeruginosa* CFU counts on the apical side were undetectable (<10^2^ CFU/well) on the wild type and CTL cell lines 72 h p.i. (Figure 5B). A few surviving PAO1 bacteria were recovered from the phage cocktail/CIP treated *cftr*- cells. One surviving colony from the combination treatment displayed a mucoid phenotype and was resistant to phage 24, but remained susceptible to ϕ69, ϕ54 as well as to CIP (mutant PT2426). Another surviving colony produced again the brown pigment pyomelanin (mutant PT2427) and was resistant to the three phages but not to CIP (Table 1). The phages reached similar titers on the three cell lines, which were lower by 1-2 logs in the presence of CIP (Figure 5C). With one exception, the proportion of ϕ24 and ϕ69 in the cocktail remained at the initial 1:1 ratio 72 h p.i., both in the absence and presence of host bacteria, suggesting that the two phages were not in competition with each other or for the bacterial host (Figure 5D).

### Phage-Treatment Survivors Are Less Cytotoxic

**FIGURE 6:**
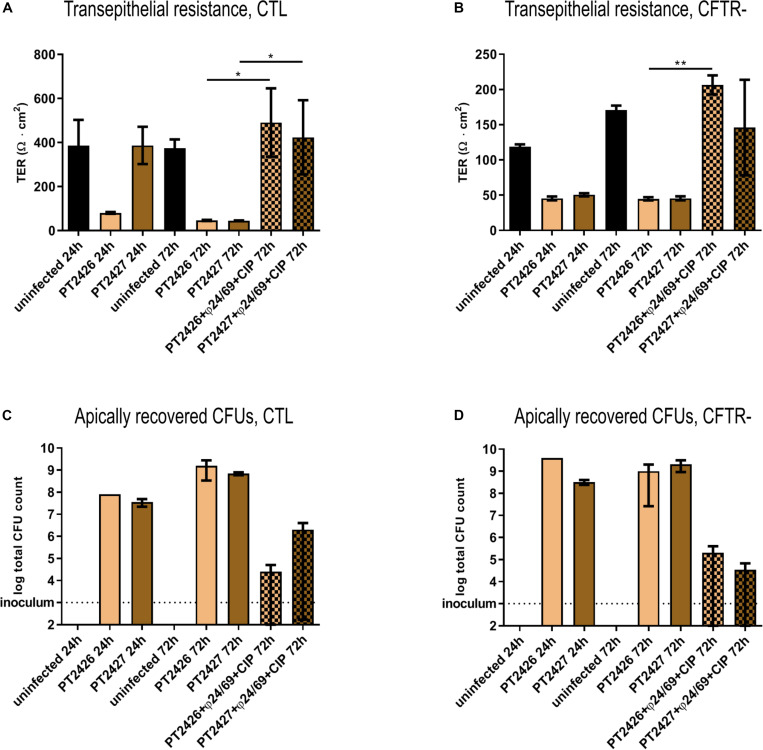
Cytotoxicity of bacteria surviving phage treatment. The cytotoxicity of PAO1-derivatives PT2426 (mucoid) and PT2527 (pyomelanin producer) were tested in absence and presence of phage cocktail/CIP treatment at 24 and 72 h p.i. on CTL **(A)** and *cftr-* cells **(B)**. The bacterial load at the apical side of CTL **(C)** and *cftr-* cells **(D)** was determined by plate counting on LB-agar plates at 24 h and 72 h p.i. Values represent mean and SEM (panel **A,B**), and median and range (panels **C,D**) of three independently performed experiments. Differences in TER between the conditions were assessed using a two-tailed *t*-test; ^∗^*p* < 0.05, ^∗∗^*p* < 0.01.

To further characterize the two clones that survived the combined phage/CIP treatment of the *cftr*- cells (Figure 5B), we infected the CTL and *cftr*- cells with PT2426 and PT2427. Surprisingly, in the absence of treatment the pyomelanin producing mutant PT2427 did not affect epithelial cell integrity of the CTL cell line 24 h p.i., but decreased eventually TER at 72 h p.i. (Figures 6A,B). The mucoid strain PT2426 showed comparable cytotoxicity as PAO1 on both the CTL and *cftr*- cell lines. However, the combined phage cocktail/CIP treatment prevented epithelial cell disruption by both clones of CTL and *cftr*- cells, suggesting that the surviving colonies were still susceptible to the cocktail/CIP combination. Indeed, at 72 h p.i the phage cocktail/CIP treatment strongly inhibited growth of PT2426 and PT2427 compared to the untreated control condition (Figures 6C,D). The results indicate that phage/antibiotic treatment does not select clones resistant to all the constituents of the cocktail and that phage-resistant clones might display attenuated virulence.

### Phage Cocktail and Ciprofloxacin Are Not Pro-inflammatory *per se*

**FIGURE 7:**
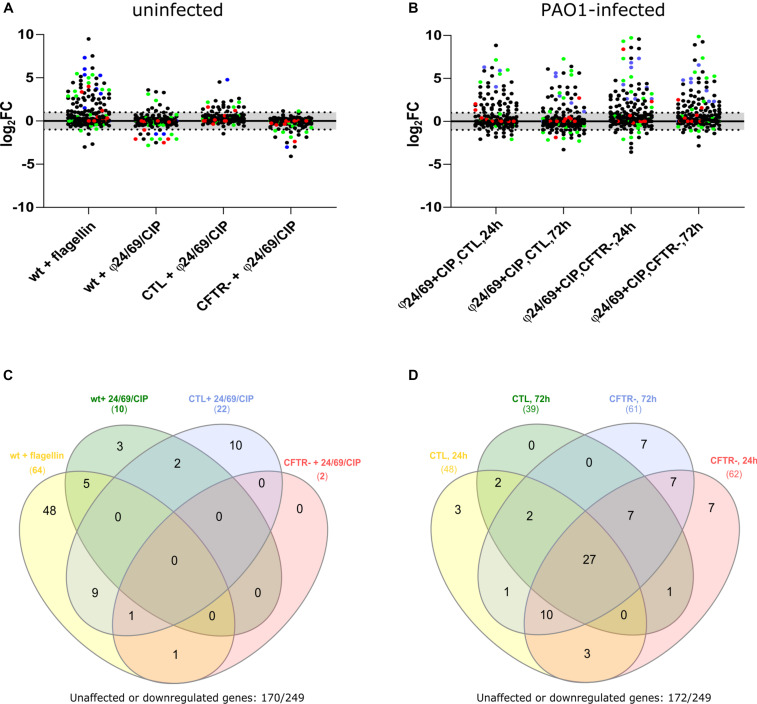
Effect of phage cocktail, ciprofloxacin (CIP) and PAO1 infection on pro-inflammatory gene expression in wild type, CTL and *cftr*-cell lines **(A)** Cells were exposed to flagellin (positive control), as well as phage cocktail/CIP combinations for 18 h. At that point total RNA was extracted from the cells on the filter, and submitted to nanoString analysis. Flagellin served as the positive control and showed induction of 64/249 genes of the nCounter Inflammation Panel. Phage cocktail in combination with CIP showed induction of only a limited number of genes by the three cell lines **(B)** Infection of cells and subsequent treatment with phage cocktail/CIP showed induction of 39–61 genes among the CTL and *cftr-* cell lines, **(C)** Venn Diagram showing upregulated genes 24 h post-treatment in the absence of PAO1. The positive control flagellin showed specific induction of 48/249 genes. None of these 64 genes was up-regulated in the three cell lines by the presence of phage cocktail/CIP. **(D)** Venn Diagram showing upregulated genes after 24 and 72 h treatments of PAO1 infected CTL and *cftr*-cell lines. *cftr*-cells showed a broader pro-inflammatory response (induction of 61 (24 h) and 62 (72 h) genes), compared to the CTL cell line (induction of 39 (24 h) and 48 (72 h) genes) in the presence of PAO1 treated with phage cocktail and CIP.

To assess whether phages alone or in combination with ciprofloxacin would induce a pro-inflammatory response in Calu-3 cells, we extracted total RNA from cells exposed for to either ciprofloxacin alone (4 mg/L), to the phage cocktail ϕ24/ϕ69 or to the combination of both. We measured the induction of pro-inflammatory genes using nano-string multiplex gene expression analysis with the nCounter^®^ Inflammation Panel (Human v2, nanoString Technologies, United States). Since untreated PAO1 infection destroyed the epithelial cell layer within 24 h, we could not extract RNA under this condition. We therefore used purified Pseudomonas flagellin, known to induce a TLR5-mediated inflammatory response, as a positive control (Zhang et al., 2005; Raoust et al., 2009). Indeed, the addition of 10 μl flagellin (1 μg/ml) at the apical side, induced 64 out of the 249 genes detectable by the Inflammation Panel (>two-fold induction) (Figures 7A,C and Supplementary Table S1). The flagellin deficient *fliC* mutant of PAO1 showed a strongly decreased response (36 genes down-regulated) on Calu-3 wild type cells (data not shown). The combination of phage and ciprofloxacin induced only ten genes in the wild type and the CTL cells, while no induction occurred in the *cftr*- cells compared to unexposed cells (Figure 7A). We therefore studied the response of CTL and *cftr*- cells infected with PAO1 and treated with phage and ciprofloxacin compared with the uninfected control. Whatever the cell line (CTL or *cftr*-), PAO1 infection induced a strong induction of pro-inflammatory genes (Figures 7A,D; Supplementary Table S2). In the CTL cell line 39 (at 24 h) and 48 (at 72 h) genes were up-regulated while 61 (at 24 h) and 62 (at 72 h) genes were up-regulated in the *cftr*- cells (Figures 7B,D). These data suggest that phage particles alone or in combination with ciprofloxacin do not induce a pro-inflammatory response, whereas flagellin and the bacterial cell lysates trigger a strong pro-inflammatory response. In fact, flagellin induced 26 out of the 27 genes up-regulated in the phage-treated PAO1 cells, suggesting that flagellin is the major trigger of the immune response to *P. aeruginosa* in epithelial cells.

## Discussion

With the increase in emergence and spread of MDR bacterial pathogens, new treatment options are urgently needed. One alternative approach is the use of bacteriophages, since their mode of action is usually not compromised by classical antibiotic resistance mechanisms. CF-patients, who are often colonized or infected by MDR or extensive drug resistant *P. aeruginosa* isolates due to repeated antibiotic courses, are a prioritized patient population for future phage trials (Morello et al., 2011; Trend et al., 2017; Rossitto et al., 2018). Here we investigated the efficacy of phages alone or in combination with an antibiotic to treat wild type as well as *cftr*- epithelial cell lines infected by *P. aeruginosa*. To our knowledge, this is the first report on phage treatment of a *cftr*- epithelial cell line. Our results show that (i) either single phage or ciprofloxacin administration maintained epithelial cell integrity upon *P. aeruginosa* infection for 24 h, (ii) only combined phage cocktail/ciprofloxacin treatment protected wild type and *cftr*- epithelial cells from *P. aeruginosa* infection during 72 h, (iii) specific mutants surviving phage treatments were less cytotoxic or more susceptible to certain antibiotics, and (iv) phage cocktails alone or in combination with ciprofloxacin *per se* did not induce a pro-inflammatory response in epithelial cells.

In agreement with other *in vitro* studies, we observed a rapid decrease in bacterial loads upon phage treatment at 24 h p.i. (Knezevic et al., 2013; Forti et al., 2018). In contrast to previous studies, the Transwell filter assay allowed us to investigate phage/bacteria/host interactions for up to 72 h. While phage or ciprofloxacin alone could not prevent bacterial regrowth after 24 h, a combined two-phage cocktail/ciprofloxacin treatment decreased *P. aeruginosa* counts by 6-logs at 72 h. Synergistic effects of antibiotic/phage combinations have been reported against *P. aeruginosa* during short (24 h) and long-term (7 days) exposures, including quinolones, beta-lactams and aminoglycosides (Knezevic et al., 2013; Torres-Barcelo et al., 2016). However, synergy seems to be phage-dependent (Uchiyama et al., 2018), and influenced by the order of phage/antibiotic administration (Chaudhry et al., 2017). Phage/antibiotic synergism is not restricted to *P. aeruginosa* but has been reported in other bacterial species (Zhang and Buckling, 2012; Torres-Barcelo and Hochberg, 2016). The underlying mechanism for this synergy is not completely understood, but could be due to antibiotic-induced cell elongation thereby increasing the number of cell associated receptors per cell, or to increased burst size mediated by induction of the SOS response (Knezevic et al., 2013; Kim et al., 2018). Phage and ciprofloxacin also synergized in a rat endocarditis model, where *P. aeruginosa* was eradicated from infected heart valves only when ciprofloxacin was co-administered with phages (Oechslin et al., 2017). One advantage of phage/antibiotic synergism is the reduction of antibiotic dosage close or below the MIC values. However, in our Calu-3 model, the high concentration of ciprofloxacin (4 mg/L) required to obtain at least a 4-log reduction in *P. aeruginosa* CFU was probably due to the uptake of ciprofloxacin by the epithelial cells. Indeed, ciprofloxacin both diffuses passively and is taken up actively by Calu-3 cells (Cavet et al., 1997; Ong et al., 2013), lowering the effective ciprofloxacin concentration on the apical side. We found that in the absence of bacteria, phages maintained their initial titers for 72 h at the apical side of the cells. Whether phages adsorb, enter and transcytose epithelial cells was not investigated here. We recovered viable phages from the basolateral side of the epithelium only when bacteria were detected concomitantly (data not shown), suggesting that phages may cross the epithelial barrier once *P. aeruginosa* has caused localized breaches by paracellular migration (Losa et al., 2014), but unlikely from transcytosis, as reported in one study (Nguyen et al., 2017). However, the study by Nguyen et al. used a different cell line and higher phage titers under slightly acidic conditions, which could explain this discrepancy.

Whether *in vitro* or *ex vivo*, we observed mutants surviving phage treatment. A frequently identified mutant population produced the brown pigment pyomelanin, secreted by *P. aeruginosa* isolates deficient in the *hmgA* gene, encoding homogentisate-1,2-dioxygenase. On the *P. aeruginosa* chromosome this gene localizes close to the *galU* gene, required for LPS synthesis, and to the *mexXY* efflux pump operon (Ramos-Aires et al., 1999; Hocquet et al., 2016). These three loci are frequently lost together as part of large chromosomal deletions (20–250 kb), occurring mainly in isolates from CF-patients (Hocquet et al., 2016). The deletion of a 40-kb DNA fragment encompassing *mexXY* explains the hyper-susceptibility of PAO1-R1.1 to ciprofloxacin and azithromycin. Another *in vitro* selected phage-resistant mutant harbored a frame shift in the *algC* gene, also required for LPS and Psl polysaccharide synthesis (Hocquet et al., 2016; Shen et al., 2018). Interestingly, a mutant surviving the phage cocktail treatment was resistant to one of the two phages but not to the other (Table 1), suggesting that careful selection of phage combinations, preferentially with two completely different targets, should reduce the risk of resistance selection. LPS mutants are less virulent in animal models and do not solicit a strong immune reaction (Goldberg et al., 1995). Indeed, the *galU* mutant was less cytotoxic than the wild-type on the CTL cell lines. Hence, phage resistance can have unexpected collateral effects, which might be detrimental to the host bacteria and impair its virulence (LPS mutants) or its intrinsic antibiotic resistance (*mexXY* deletion). An interesting exploitation of such collateral effects was the recently reported identification of a phage targeting the outer membrane protein OprM of the MexAB-OprM multidrug efflux pump of *P. aeruginosa* (Chan et al., 2016). Mutants resistant to this phage through loss or inactivation of the OprM protein were hyper-susceptible to antibiotics and would therefore be more responsive to antibiotic treatments than their phage susceptible wild-type parents (Chan et al., 2016).

An important issue for future phage treatments is the survival and innocuity of phage particles. Our data showed that even after remaining 72 h in the air-liquid interface without active replication, phage particles were not degraded and remained infective. We further observed that neither phage nor ciprofloxacin alone or in combination solicited a pro-inflammatory response in Calu-3 wild type or *cftr*- cells, a result in agreement with *in vivo* data (Roach et al., 2017). Whether phages would increase or suppress the inflammatory response due to phage-induced lysis of *P. aeruginosa* could not be clearly established. However, we found that the majority of pro-inflammatory genes up-regulated in Calu-3 cells by flagellin were also induced by phage or antibiotic-treated *P. aeruginosa*, suggesting that flagellin recognition by TLR-5 is the major signal stimulating a host immune response (Zhang et al., 2005; Parker and Prince, 2013). Our *ex vivo* model did not include host immune cells like macrophages and neutrophils, which assist antibiotics or phages to clear bacterial infections in the host (Oechslin et al., 2017; Roach et al., 2017). Furthermore, binding of phage to host proteins as well as cellular and antibody responses and accessibility of the infection site likely affect phage survival and treatment efficacy. However, our model would be representative of patients with a compromised immune system (chronic granulomatous disease) or transplant-patients receiving life-long immune-suppressive therapies.

In conclusion, our data showed the requirement of phage cocktail/antibiotic combinations to decrease the bacterial load in order to avoid the emergence of resistance. Furthermore, rational design or selection of phages recognizing essential bacterial targets should be a promising avenue to optimize future phage therapies.

## Data Availability Statement

The original contributions presented in the study are publicly available. This data can be found here: http://www.ncbi.nlm.nih.gov/bioproject/648055.

## Author Contributions

AL, JS, and LF performed the cell culture and *in vitro* experiments. GR provided phages and performed initial phage screening. MC, AL, JS, CD, and TK designed the experiments and analyzed the data. BV and DH performed the bioinformatics analyses. TK wrote the manuscript. All the authors reviewed and approved the manuscript.

## Conflict of Interest

The authors declare that the research was conducted in the absence of any commercial or financial relationships that could be construed as a potential conflict of interest.
